# p75 neurotrophin receptor: a prototype therapeutic target modulating fundamental mechanisms of degeneration and glial function in Alzheimer’s and related dementias

**DOI:** 10.1002/alz70859_098673

**Published:** 2025-12-25

**Authors:** Frank Longo

**Affiliations:** ^1^ Stanford University, Stanford, CA USA

## Abstract

**Background:**

In AD and ADRDs, multiple pathological processes including those promoted by amyloid, pathological forms of tau and dysfunction of glial and innate immunity functions contribute to the functional failure and degeneration of synapses. The p75 neurotrophin receptor (p75NTR) is expressed by neurons and glial populations involved in AD/ADRDs and in its primary functional role, promotes degenerative signaling, including many of the signaling modules that contribute to synaptic degeneration. LM11A‐31 is an orally CNS bioavailable small molecule p75NTR modulator that downregulates p75NTR degenerative signaling and upregulates its trophic signaling.

**Method:**

The effects of small molecule modulation of p75NTR on neuronal and glial processes promoting synaptic degeneration have been characterized by the application of LM11A‐31 to in vitro neuronal and glial models involving exposure to amyloid‐beta oligomers or tau oligomers; and application to both APP‐based (APP‐Lon/Swe) and tau‐based (PS19) mouse models. Outcome measures include signaling, mitochondrial, RNAseq, morphological, synaptic, behavioral and biomarkers.

**Result:**

Small molecule modulation of p75NTR counteracts degenerative signaling promoted by amyloid‐, tau‐ and glial‐related mechanisms. This effect is associated with a reduction in the following: morphological measures of synaptic degeneration, accumulation and spreading of pathological tau species, glial abnormalities, loss of LTP in hippocampal slice studies and loss of behavioral measures. Treatment is also associated with a reduction in brain accumulation of p‐tau217. Alternations in AD‐related transcriptomic signature modules, including those in huma data sets, were substantially normalized.

**Conclusion:**

These findings support the hypothesis that modulation of p75NTR signaling, a key mechanism and hub upstream from a broad network of AD/ADRD degenerative signaling networks, leads to a reduction of synaptic and glial pathology. The transcriptomic findings indicate some degree of overlap between the mouse findings and human AD degenerative mechanisms. This overlap and these preclinical findings are consistent with biomarker and proteomic findings observed in a recent phase 2a clinical trial of LM11A‐31 with mild‐moderate AD participants.

